# Hemoglobinopathy: Molecular Epidemiological Characteristics and Health Effects on Hakka People in the Meizhou Region, Southern China

**DOI:** 10.1371/journal.pone.0055024

**Published:** 2013-02-01

**Authors:** Min Lin, Ying-Fang Wen, Jiao-Ren Wu, Qian Wang, Lei Zheng, Gui-Rong Liu, Yue Huang, Hui Yang, Fen Lin, Xiao-Fen Zhan, Chun-Ping Lin, Hui-Tian Yang, Qiu-Qing Weng, Fen-Ting Huang, Yuan Wang, Mei-Qiong Yao, Hui-Zhou Chen, Di-Hong Wu, Jing-Bo Zeng, Ri-Xin Zeng, Hua Yang, Gui-Cai Li, Min Lu, Juan-Juan Zhu, Long-Xu Xie, Jun-Li Wang, Li-Ye Yang

**Affiliations:** 1 Laboratory Medical Center, Chaozhou Central Hospital Affiliated to Southern Medical University, Chaozhou, Guangdong Province, People’s Republic of China; 2 Medical Laboratory, Meixian People’s Hospital, Meizhou, Guangdong Province, People’s Republic of China; 3 Laboratory Medical Center, Nanfang Hospital, Southern Medical University, Guangzhou, Guangdong Province, People’s Republic of China; 4 Medical Laboratory, Jiaoling People’s Hospital, Meizhou, Guangdong Province, People’s Republic of China; 5 Medical Laboratory, Pingyuan Women and Children Hospital, Meizhou, Guangdong Province, People’s Republic of China; 6 Medical Laboratory, Fengshun People’s Hospital, Meizhou, Guangdong Province, People’s Republic of China; 7 Medical Laboratory, Dabu People’s Hospital, Meizhou, Guangdong Province, People’s Republic of China; 8 Medical Laboratory, Xingning People’s Hospital, Meizhou, Guangdong Province, People’s Republic of China; 9 Medical Laboratory, Wuhua People’s Hospital, Meizhou, Guangdong Province, People’s Republic of China; 10 Department of Health Examination, Meizhou Entry-Exit Inspection and Quarantine Bureau, Meizhou, Guangdong Province, People’s Republic of China; 11 Chaozhou Hybribio Limited Corporation, Chaozhou, Guangdong Province, People’s Republic of China; 12 Center of Clinical Laboratory, Affiliated Hospital of Youjiang Medical College for Nationalities, Baise, Guangxi, China; Gentofte University Hospital, Denmark

## Abstract

**Background:**

Hemoglobinopathies are the most common inherited diseases in southern China. However, there have been only a few epidemiological studies of hemoglobinopathies in Guangdong province.

**Materials and Methods:**

Peripheral blood samples were collected from 15299 “healthy” unrelated subjects of dominantly ethnic Hakka in the Meizhou region, on which hemoglobin electrophoresis and routine blood tests were performed. Suspected cases with hemoglobin variants and *hereditary persistence of fetal hemoglobin* (HPFH) were further characterized by PCR, DNA sequencing, reverse dot blot (RDB) or multiplex ligation-dependent probe amplification (MLPA). In addition, 1743 samples were randomly selected from the 15299 subjects for thalassemia screening, and suspected thalassemia carriers were identified by PCR and RDB.

**Results:**

The gene frequency of hemoglobin variants was 0.477% (73/15299). The five main subgroups of the ten hemoglobin variants were Hb E, Hb G-Chinese, Hb Q-Tahiland, Hb New York and Hb J-Bangkok. 277 cases (15.89%, 277/1743) of suspected thalassemia carriers with microcytosis (MCV<82 fl) were found by thalassemia screening, and were tested by a RDB gene chip to reveal a total of 196 mutant chromosomes: including 124 α-thalassemia mutant chromosomes and 72 β-thalassemia mutant chromosomes. These results give a heterozygote frequency of 11.24% for common α and β thalassemia in the Hakka population in the Meizhou region. 3 cases of HPFH/δβ-thalassemia were found, including 2 cases of Vietnamese HPFH (FPFH-7) and a rare Belgian^ G^γ(^A^γδβ)^0^–thalassemia identified in Chinese.

**Conclusions:**

Our results provide a detailed prevalence and molecular characterization of hemoglobinopathies in Hakka people of the Meizhou region. The estimated numbers of pregnancies each year in the Meizhou region, in which the fetus would be at risk for β thalassemia major or intermedia, Bart’s hydrops fetalis, and Hb H disease, are 25 (95% CI, 15 to 38), 40 (95% CI, 26 to 57), and 15 (95% CI, 8 to 23), respectively.

## Introduction

Hemoglobinopathy is a kind of genetic defects that result in abnormal structure of one of the globins [1] and, in most cases, is inherited as autosomal co-dominant traits [2]. The hemoglobinopathies encompass all genetic diseases of hemoglobin. They fall into two main groups: thalassemia syndromes and structural hemoglobin variants (abnormal hemoglobins) [1], [3]. Only a few hemoglobin variants show the hematological phenotype of thalassemia, such as abnormal hemoglobin E (Hb E) [4]. Another heterogeneous group of related Hb disorders, hereditary persistence of fetal hemoglobin (HPFH), is usually caused by mutations in the *β*-globin gene cluster, lead to reduced expression of certain types of globin genes and is usually classified into ‘the thalassemia syndromes’. The condition is usually asymptomatic, and is only noticed when screening for other hemoglobin disorders [5], [6]. The variable clinical manifestations of hemoglobinopathy range from normal to severe, transfusion-dependent anemia. The frequency of hemoglobinopathies varies considerably with both geographical location and ethnic group. Hemoglobinopathy is common in various ethnic populations from Africa, the Mediterranean area and southeast Asia, including southern China, and creates a major public health problem and social burden in these regions owing to their high prevalence and incidence [7], [8].

**Figure 1:**
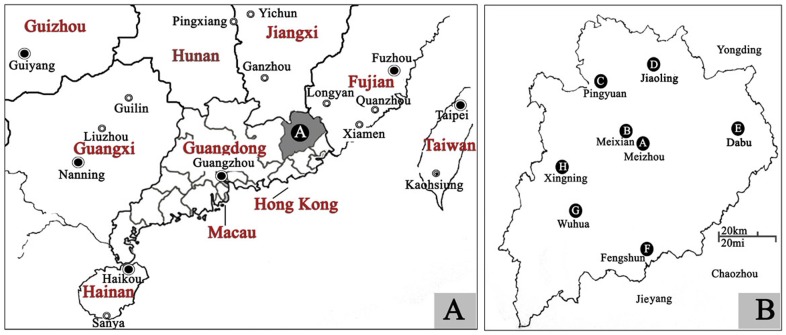
Geographic location of the Meizhou region and its surrounding areas. **A:** The Meizhou region in southern China. **B:** The eight areas in the Meizhou region investigated in this study: Meizhou urban (A)/Meixian (B), Pingyuan(C)/Jiaoling(D) (north), Dabu (E) (west), Fengshun (F) (south) and Xingning (H)/Wuhua (G) (east).

With a total area of 15,836 km^2^ and a population of 5.05 million, the Meizhou region is located in the northeast of Guangdong Province, P. R. China. It borders Fujian Province to northeast and Jiangxi Province to northwest (Figure-1A). More than 95% people lived in Meizhou are Hakka. Hakka is a intriguing Han Chinese populations that mainly inhabit southern China, which is characteristic of their unique culture and is distinct from the traditional culture of southern Hans (SHs), but show lots of similarities to that of northern Hans (NHs), including some features in dialects, life styles, customs, and habits [9], [10]. For instance, the famous architectural type of Hakka, Round House (Called Weiwu in Chinese), is suggested to be derived from NH’s quadrate yard (Called Siheyuan in Chinese) [9]. Hitherto, Hakka have been studied using different genetic markers, e.g. classical markers, autosomal microsatellites/SNPs, and Y chromosome SNP, most of which preferred the northern origins of Hakka and/or Chaoshanese [9], [10]. Guangdong Province contains the largest Hakka community, particularly in the so-called Xing-Mei (Xingning-Meizhou) area. Currently, few data are available on the prevalence and molecular characterization of hemoglobinopathies for the Hakka population of the Meizhou region.

In this study, we perform a large-scale survey of hemoglobinopathies (hemoglobin variants, thalassemia and HPFH) in 15299 “healthy” subjects from Hakka in the Meizhou region, which allows us to document the prevalence and molecular characterization of hemoglobinopathies in this region. In addition, we report a case of ^G^γ(^A^γδβ)^0^ -thalassemia mutation and a rare hemoglobin variant found in Chinese for the first time.

## Materials and Methods

### Population Samples

**Figure 2:**
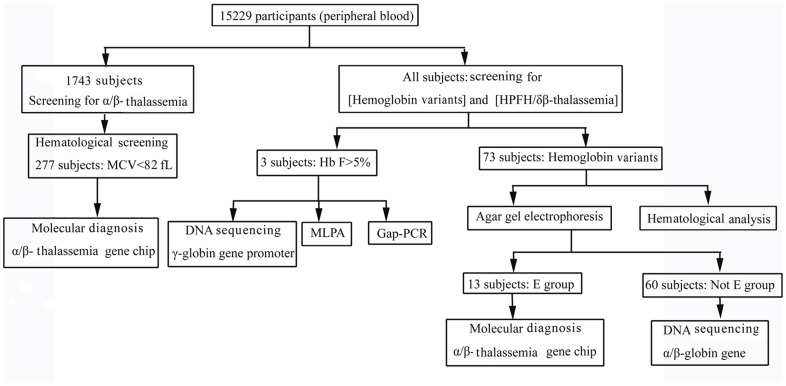
Diagram for the screening of hemoglobin variants, α/β-thalassemia and HPFH/*δβ*-thalassemia.

The study population included 15299 unrelated “healthy” subjects for hemoglobinopathy screening between February 2011 and June 2011. These subjects visited medical units for routine healthy examination including blood tests, and the discarded blood samples were used for further study. The ages of these subjects ranged from 5 to 70-year-old and about 95% were Meizhou aborigines, i.e., Hakka people. Information sheets with nationality, sex, age, dialect, Meizhou aborigines or not and written consent forms were available in Chinese to ensure comprehensive understanding of the study objectives, and informed consent was signed or thumbprinted by the participants or their guardians. All studies were approved by the Ethics Committee of Meixian People’s Hospital and Ethics Committee of Chaozhou Central Hospital Affiliated to Southern Medical University. Figures 1A and B show the location of Meizhou and the eight study regions, respectively. These regions include Meizhou urban (A)/Meixian (B) (middle, 8772 subjects), Pingyuan(C)/Jiaoling(D) (north, 2086 subjects), Dabu (E) (west, 1100 subjects), Fengshun (F) (south, 1042 subjects) and Xingning (H)/Wuhua (G) (east, 2299 subjects) (Figure 1B). These subjects received screening for the presence of hemoglobinopathy in the Chaozhou Centeral Hospital. The flowchart of screening strategy used in this study is illustrated in Figure 2.

### Hematological Analysis

2 mL peripheral blood samples were taken from all subjects and routine blood tests were performed and sent (at 4°C) to the Hematology and Molecular Laboratory for further analysis. [i] All subjects were analyzed by standard cellulose acetate electrophoresis at pH = 8.6 for hemoglobin variants and HPFH/δβ-thalassemia. The results of electrophoresis were classified as the ‘fast’ hemoglobin and ‘slow’ hemoglobin, including hemoglobin H, J, K, Bart’s, Normal, F/Q, G/D and E. The percentages of variants and Hb F were measured by traditional methods as our previous report [11]. Agar gel electrophoresis was used to further analyze all hemoglobin variants by using Helena Spife 3000 (Helena). Samples with Hb F >5% were considered for possible HPFH/δβ-thalassemia carriers; [ii] 1743 samples were randomly selected from 15299 subjects for thalassemia screening. Hematological data of these 1743 subjects were collected on an automated blood cell counter (Sysmex XT-1800i; Sysmex Corporation, Japan). Samples with MCV values of <82.0 fL were considered as possible thalassemia carriers.

### Molecular Diagnosis of α/β-Thalassemia

Genomic DNA of subjects with MCV <82.0 fL, subjects with hemoglobin variants and subjects with Hb F values of >5% were extracted from peripheral blood leukocytes by DNA blood mini kit **(**QIAGEN China Shanghai Co., Ltd). The DNA concentration was determined by UV spectrophotometer (UNICO Shanghai Instruments Co., Ltd) at the wavelength of 260 nm. The three known α-thalassemia deletions –^SEA^, -α^3.7^, -α^4.2^, two α-thalassemia mutations [Hb Constant Spring (NM_000517.4:c.427T>C) and Hb Quong Sze (NM_000517.4:c.377T>C)] and 16 known β-thalassemia mutations most commonly seen in the Chinese population [−29M(A→G) (HBB:c.−79A>G), −28M(A→G) (HBB:c. –78A>G), CapM(-AAAC) (HBB:c.−50A>C), IntM(T→G) (HBB:c.2T>G), 14/15M(+G) (HBB:c.45_46insG), 17M(A→T) (HBB:c.52A>T), βeM(G→A) (HBB:c.79G>A), 27/28M(+C) (HBB:c.84_85insC), 31M(-C) (HBB:c.94delC), IvsI-1M(G→A, G→T) (HBB:c.92+1G>T and HBB:c.92+1G>A), IvsI-5M(G→C)(HBB:c.92+5G>C), 41/42M(-TTCT) (HBB:c.126_129delCTTT), 43M(G→T) (HBB:c.130G>T), 71/72M(+A) (HBB:c.216_217insA), 654M(C→T) (HBB:c.316-197C>T)] were analyzed by a thalassemia gene chip (Chaozhou Hybribio Limited Corporation, China). The kit of the gene chip was approved by Chinese SFDA (REG.NO: SFDA (P)20123400399). The primers and probes were described in our previous report [12]. The assay was performed according to the manufacturer’s protocol.

### DNA Sequencing for Hb Variants

The α_1_ and α_2_ globin genes were amplified by PCR and sequenced. The optimized hotstart amplification reaction system with a total volume of 50 µl including 100 ng of genomic DNA, 25 pmol of forward and reverse primers, 1 mM MgCl_2_, 200 µM of each dNTP, 2.5 U LA Taq polymerase (TaKaRa, Dalian, China) in 1 × dimethyl sulfoxide (DMSO) buffer (32 mM (NH_4_)_2_SO_4_, 134 mM Tris-HCl at pH 8.8, 20% DMSO and 20 mM β-mercaptoethanol) with 0.7 M betaine. Reactions were carried out in an MJ Mini Personal Thermal Cycler (Bio-RAD Company) with an initial denaturation step of 95°C for 10 min and then 35 cycles of 95°C for 1 min, 58°C for 1 min, 72°C for 1.5 min with a final extension at 72°C for 10 min. The α_2_ (880 bp) and α_1_ (880 bp) gene fragments were sequenced by an ABI 377 automated sequencer (Applied Biosystems, Foster City, CA) with the same primers in Table 1 [11].

**Table 1:** α, β, ^A^γ and ^G^γ gene globin primers for PCR and sequencing.

Name	Gene	Sequencing (5′-3′)	Product size (bp)
L	α_2_ gene	TCCCCACAGACTCAGAGAGAACC	880
D		AACACCTCCATTGTTGGCACATTCC	
L	α_1_ gene	TCCCCACAGACTCAGAGAGAACC	880
A		CCATGCCTGGCACGTTTGCTGAG	
B1	β gene(exon1-2)	AAGGCTGGATTATTCTGAGTC	700
B2		TGTATTTTCCCAAGGTTTGA	
B3	β gene (exon 3)	CTAGGGTTGGCCAATCTACTC	446
B4		GCAATCATTCGTCTGTTTCC	
^A^γ-FP	^A^γ gene	TGAAACTGTTGCTTTATAGGAT	658
^A^γ-RP		GAGCTTATTGATAACCTCAGACG	
^G^γ-FP	^G^γ gene	CTGCTAACTGAAGAGACTAAGATT	723
^G^γ-RP		CAAATCCTGAGAAGCGACCT	

DNA sequencing of the β-globin gene was performed using the primers shown in Table 1, and DNA was amplified using Taq polymerase (TaKaRa, Dalian, China) in MJ Mini Personal Thermal Cycler (Bio-RAD Company). After initial denaturation at 95°C for 3 min, 35 cycles of PCR (95°C for 30 s, 57°C for 30 s and 72°C for 1 min) were performed. The primers and product lengths were shown in Table 1 [11]. DNA sequencing was performed by the ABI 3700 automated sequencer.

### PCR and DNA Sequencing for Common HPFH/δβ-thalassemia in Chinese

Gap-PCR was used to identify three deletion genotypes of HPFH/δβ-thalassemia including Chinese (^A^γδβ)^0^ thalassemia, Southeast Asian (Vietnamese) deletion and Thai (^A^γδβ)^0^ thalassemia (HPFH-6), which was the commonest genotypes in China [13], [14]. The primers and reaction system were performed as previous reports [14]–[15].

DNA sequencing of the ^A^γ promoter and ^G^γ promoter were performed to diagnose two common undeletional genotypes, which included ^A^γ-HPFH -196(C>T) and ^G^γ^A^γδβ-thalassemia −29 (A>G) [6], [16]. The primers were shown in Table 1, and DNA was amplified using Taq polymerase (TaKaRa, Dalian, China) in MJ Mini Personal Thermal Cycler (Bio-RAD Company). After initial denaturation at 95°C for 3 min, 35 cycles of PCR (94°C for 60 s, 66°C for 30 s and 72°C for 1 min) were performed. DNA sequencing was performed by the ABI 3700 automated sequencer with the same primers.

### MLPA for HPFH/δβ-thalassemia

Gene dosage analysis of the LCR region and HBE, HBG2, HBG1, HBD and HBB on the β-globin gene cluster was performed with the SALSA MLPA KIT P102 HBB kit (MRC Holland, Amsterdam, the Netherlands) according to the manufacturer’s instructions [14]. The multiplex ligation-dependent probe amplification analysis (MLPA) consisted of approximately 100 ng of genomic DNA. Ligation and amplification were carried out on an MJ Mini Personal Thermal Cycler (Bio-RAD Company). The polymerase chain reaction (PCR) conditions included 35 cycles of 95°C for 30 s, 60°C for 30°C s and 72°C for 60°C s, followed by a final extension at 72°C for 20 min. All amplified fragments were separated using capillary electrophoresis on an ABI PRISM 3130 Genetic Analyzer (Applied Biosystems). The area under the peak for each amplified fragment was measured and normalized in comparison with the peak areas of normal control individuals using GeneMarker software v.1.8 (Soft-Genetics, State College, PA, USA). Threshold ratios for deletion and duplication were set at <0.6 and >1.3, respectively.

### Statistical Analysis

Statistical analysis were conducted with SPSS 16.0 statistical software. The prevalence of different hemoglobinopathy alleles was calculated from the standard Hardy-Weinberg formula. Data from the Meizhou region and previous studies were analyzed by Pearson χ^2^ test with Bonferroni adjustment for multiple testing. *P*<0.05 was considered statistically different.

## Results

### Hemoglobin Variants in Meizhou Region

In this survey, 73 cases of hemoglobin variants were found by hemoglobin electrophoresis, including 33 cases of α-chain variants and 40 cases of β-chain variants. The gene frequency of abnormal Hbs was 0.477% (73/15299) in the Meizhou region. All these alleles were found in Hardy-Weinberg equilibrium (α-alleles:*P* = 0.894; β-alleles:*P* = 0.871), the gene mutation frequencies of α-globin chain and β-globin chain were 1.08 ×10^−3^ and 1.31 ×10^−3^, respectively.

**Figure 3:**
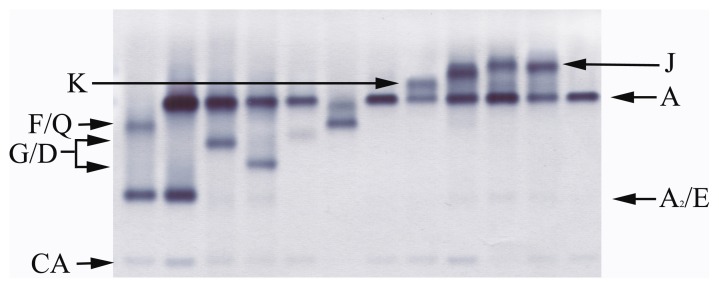
Hemoglobin analysis of hemoglobin variants with cellulose acetate electrophoresis at pH = 8.6. The electrophoresis phenomenon includes H, J, K, Normal, F/Q, G/D and E.

According to the phenotype of hemoglobin electrophoresis, these 73 cases of abnormal hemoglobin could be divided into five groups (J, K, Q, G/D and E) as previous report [11]. The G/D (26.7%, 18/73) and E groups (26.7%, 18/73) were the main types of abnormal hemoglobin in the Meizhou region, and followed by the Q group (17.8%, 13/73) and K group (16.4%, 12/73). The hemoglobin electrophoresis of these hemoglobin variants were shown in Figure 3. This result was consistent with previous report from this region [17].

**Figure 4:**
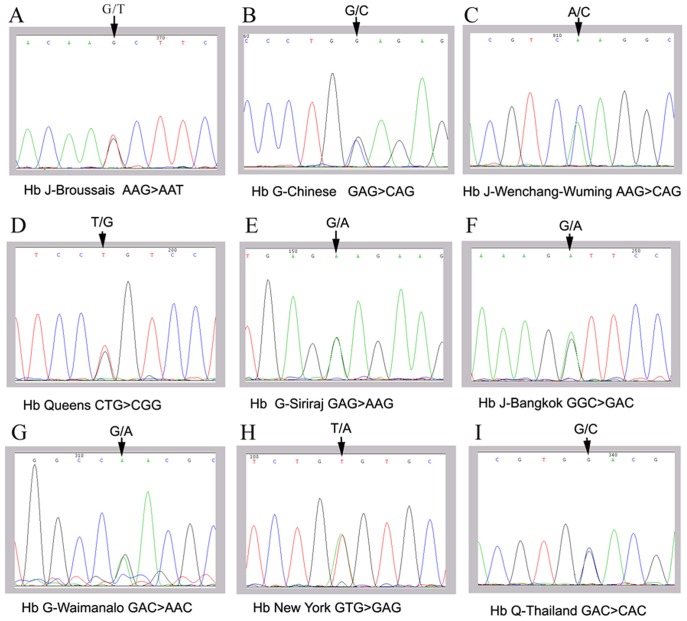
DNA sequence of 9 kinds of hemoglobin variants. A: Hb J-Broussais (HBA2:c.273G>T); **B:** Hb G-Chinese (HBA2:c.91G>C); **C:** Hb Hb J-Wenchang-Wuming (HBA2:c.34A>C); **D:** Hb Queens (HBA2:c.104T>G); **E:** Hb G-Siriraj (HBB:c.22G>A); **F:** Hb J-Bangkok (HBB:c.170G>A); **G:** Hb G-Waimanalo (HBA2:c.193G>A); **H:** Hb New York (HBB:c.341T>A); **I:** Hb Q-Thailand (HBA1:c.223G>C).

Ten hemoglobin variants were found from the Hakka population in the region (Table 2). Hb E (24.7%), Hb Q-Thailand (17.8%), Hb NewYork (16.4%) and Hb G-Chinese (16.4%) are the four main hemoglobin variants (Table 2). All 13 cases of Hb Q-Thailand were also linked with the 4.2 kb deletion. Therefore, these people show slight red cell microcytosis (data not shown), and this result is consistent with previous reports [18], [19]. In the survey, a rare case of Hb J-Broussais (Hb Tagawa-I) was found and identified, and is the first report of such hemoglobin variant in Chinese. The DNA sequencing results of 9 types of abnormal hemoglobin (except HbE) are shown in Figure 4.

**Table 2:** Hemoglobin variants among 15229 subjects from Meizhou region.

Hemoglobin Variants	HGVS name	Residue	Substitution	Ele group	No	Percentage(%)
α-globin gene mutation					33	45.2
Hb J-Broussais	HBA2:c.273G>T	90(FG2)	Lys>Asn	J	1	1.4
Hb J-Wenchang-Wuming	HBA2:c.34A>C	11(A9)	Lys>Gln		4	5.5
Hb Q-Thailand	HBA1:c.223G>C	74(EF3)	Asp>His	Q	13	17.8
Hb Queens	HBA2:c.104T>G	34(B15)	Leu>Arg	G/D	2	2.7
Hb G-Waimanalo	HBA2:c.193G>A	64(E13)	Asp>Asn		1	1.4
Hb G-Chinese	HBA2:c.91G>C	30(B11)	Glu>Gln		12	16.4
β-globin gene mutation					40	54.8
Hb J-Bangkok	HBB:c.170G>A	56(D7)	Gly>Asp	J	7	9.6
Hb New York	HBB:c.341T>A	113(G15)	Val>Glu	K	12	16.4
Hb G-Siriraj	HBB:c.22G>A	7(A4)	Glu>Lys	G/D	3	4.1
Hb E	HBB:c.79G>A	26(B8)	Glu>Lys	E	18	24.7
Total					73	100

* **Ele group:** electrophoresis group. The results of electrophoresis were classified as the ‘fast’ hemoglobin and ‘slow’ hemoglobin, including hemoglobin H, J, K, Bart’s, Normal, F, Q, G/D and E; No. : case number.

### Population Prevalence and Mutation Spectrum of α and β Thalassemia

**Figure 5:**
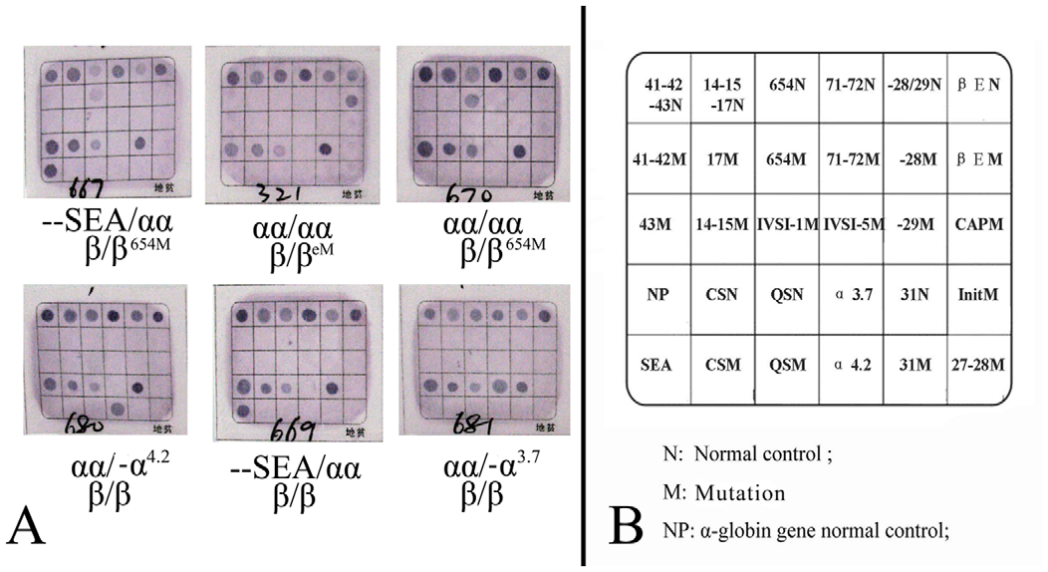
Results of α and β thalassemia in gene chip. A: The results of α and β thalassemia in the gene chip; B: The location of the probes dotted in the gene chip used for the reverse dot blot assay. Locations of wild-type and mutant probes are denoted N and M, respectively.

A total of 1743 blood samples were obtained and analyzed from the Meizhou region. 277 cases of microcytosis (MCV<82 fl) were found, yielding a very high percentage of 15.89% (i.e., 277/1743). All 277 microcytosis samples were analyzed by flow-through hybridization and a RDB gene chip for the three known α-thalassemia deletions (–SEA, -α^3.7^, -α^4.2^), two α-thalassemia mutations (Hb Constant Spring and Hb Quong Sze) and 16 known β-thalassemia mutations most commonly seen in Chinese population, positive results were observed in chips in Figure 5.

A total of 196 mutant chromosomes were identified (Table 3), including 124 α-thalassemia mutant chromosomes and 72 β-thalassemia mutant chromosomes. All these alleles were found in Hardy-Weinberg equilibrium. This result corresponds to a heterozygote frequency of 11.24% for common α and β thalassemia in the Hakka population of the Meizhou region, including 7.11% of α-thalassemia and 4.13% of β-thalassemia. Eleven samples were compound carriers of α-thalassemia and β thalassemia, giving a frequency of 0.63% (11/1743) for coincidence of these two common disorders in this population.

**Table 3:** Population prevalence and genotypes of thalassemia among 1743 blood samples from Meizhou.

Genotype	Type	No.	F (%)	CR (%)	AN	P (%)
α-thalassemia chromosomes		–	–	–	124^a^	7.11
–^SEA^/αα	alpha-1	85	4.88	46.45	–	–
-α^3.7^/αα	alpha-2	12	0.69	6.56	–	–
-α^4.2^/αα	alpha-2	8	0.46	4.37	–	–
αα^CS^/αα	HbVar	3	0.17	1.64	–	–
αα^QS^/αα	HbVar	1	0.06	0.55	–	–
–^SEA^/−α^3.7^		1	0.06	0.55	–	–
–^SEA^/−α^4.2^		1	0.06	0.55	–	–
β-thalassemia chromosomes		–	–	–	72^b^	4.13
IVSII-654(C>T)/N	beta+	23	1.32	12.57	–	–
CD41-42(-CTTT)/N	beta0	13	0.75	7.10	–	–
−28(A>G)/N	beta+	8	0.46	4.37	–	–
CD17(A>T)/N	beta0	8	0.46	4.37	–	–
CD27-28(+C)/N	beta0	3	0.17	1.64	–	–
βeM(G>A)	HbVar	2	0.11	1.09	–	–
−29(A>G)/N	beta+	1	0.06	0.55	–	–
CD43(G>T)/N	beta0	1	0.06	0.55	–	–
CD14-15(+G)/N	beta0	1	0.06	0.55	–	–
IVSI-5(G>C)/N	beta+	1	0.06	0.55	–	–
–^SEA^/αα and CD41-42(-CTTT)/N		2	0.11	1.09	–	–
–^SEA^/αα and CD17(A>T)/N		1	0.06	0.55	–	–
–^SEA^/αα and -28(A>G)/N		1	0.06	0.55	–	–
-α^3.7^/αα and IVSII-654(C>T)/N		4	0.23	2.19	–	–
-α^3.7^/αα and -28(A>G)/N		1	0.06	0.55	–	–
-α^4.2^/αα and IVSII-654(C>T)/N		2	0.11	1.09	–	–
Total		183	10.50	100	196	11.24

* No.: case number; F: genotype frequency; CR: constituent ratio; AN: allele number; P: percentage; ^a^These numbers are involved in the total number of α-thalassaemia alleles, including two cases of Hb H disease and 11 cases of α-thalassemia compound β-thalassemia. ^b^These numbers are involved in the total number of β-thalassemia alleles, including 11 cases of α-thalassemia compound β-thalassemia.

Consistent with previous reports [3], [20], [21], the Southeast Asian type of deletion (–SEA) is the most common α-thalassemia mutation, followed by -α^3.7^ deletion and -α^4.2^ deletion. The heterozygote frequencies for these 3 common deletions are 5.22% (91/1743), 1.03% (18/1743) and 0.63% (11/1743), respectively (data including α-thalassemia carriers, Hb H diseases and compound carriers of α-thalassemia and β thalassemia). 10 kinds of β-thalassemia genotypes were found in the molecular survey (Table 3). IVS-II-654(C→T) and CD41/42 (-TTCT) account for 50% of these mutations, again in agreement with previous studies [3], [20].

### Hereditary Persistence of Fetal Hemoglobin (HPFH)/δβ-thalassemia

**Figure 6:**
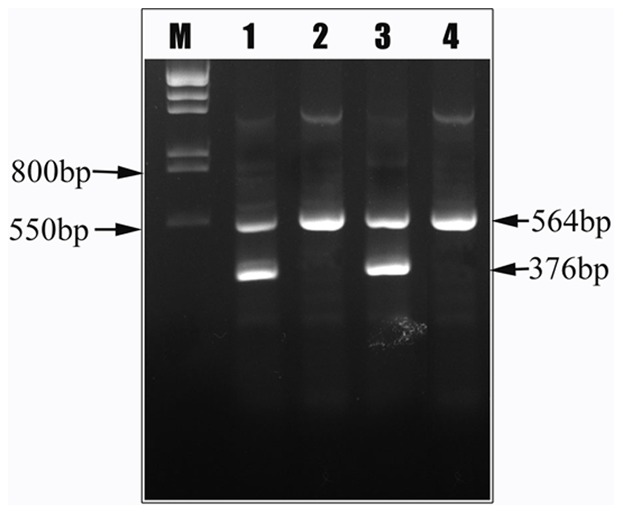
Results of 2 cases of Vietnamese HPFH (FPFH-7) were identified by Gap-PCR.

**Figure 7:**
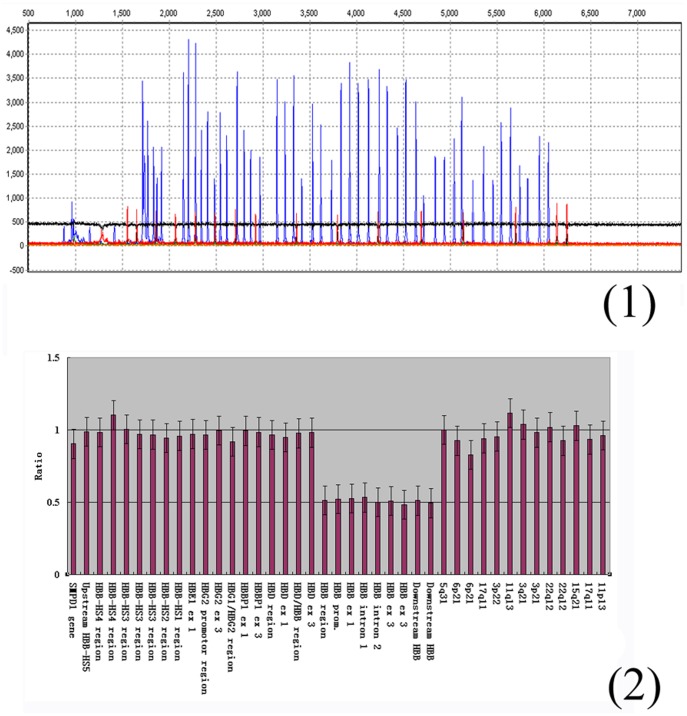
The MLPA result of Vietnamese HPFH (FPFH-7). (1) The analysis of DNA product by ABI 3730 for Vietnamese HPFH; (2) Data histograms of Vietnamese HPFH.

Three cases of HPFH/δβ-thalassemia were found by hemoglobin electrophoresis screening (pH = 8.6). The gene frequency is 0.002% (3/15299). No mutation was found in ^A^γ promoter and ^G^γ promoter by DNA sequencing from these 3 samples. 2 cases of Vietnamese HPFH (FPFH-7) were identified by Gap-PCR (Figure 6), and were also confirmed by MLPA (Figure 7). The hematological and molecular findings of 3 cases were shown in Table 4, consistent with previous reports of heterozygous state [6], [14], Vietnamese HPFH gives rise to a typical β-thalassemia trait phenotype with microcytosis and increased HbA_2_, albeit with a significantly raised Hb F level as well. It was a common HPFH in southern Chinese [6].

**Table 4:** Hematological and molecular findings of three cases of HF>5% samples.

No	RBC(10^12^/L)	Hb(g/L)	HCT (%)	MCV(fL)	Hb A(%)	Hb A_2_(%)	Hb F(%)	Genotype
M-1	5.61	135	42.0	75.0	82.5	3.9	13.6	HPFH-7; Vietnamese HPFH
M-2	5.20	122	38.7	74.5	81.2	4.3	14.5	HPFH-7; Vietnamese HPFH
M-3	6.06	116	40.0	66.0	77.9	4.9	17.2	Belgian^ G^γ(^A^γδβ)^0^ –thalassemia

* RBC, red cell count; Hb, hemoglobin; HCT, hematocrit; MCV, mean corpuscular volume; MCH, mean corpuscular hemoglobin.

**Figure 8:**
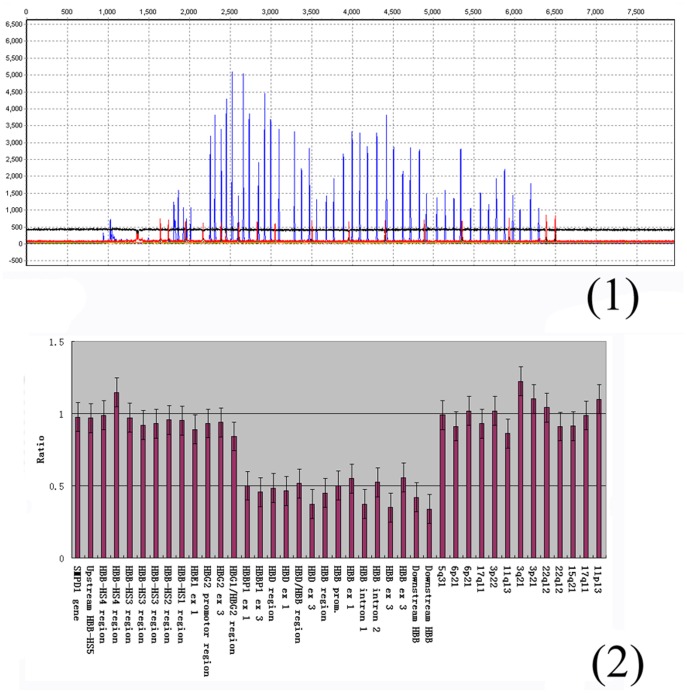
The MLPA result of Belgian^ G^γ(^A^γδβ)^0^ -thalassemia. (1) The analysis of DNA product by ABI 3730 for Belgian^ G^γ(^A^γδβ)^0^ -thalassemia; (2) Data histograms of Belgian^ G^γ(^A^γδβ)^0^ -thalassemia.

A rare Belgian^ G^γ(^A^γδβ)^0^ -thalassemia was found in the screening. Based on MLPA results of this sample, gene copy number reduction from probe 06400-L05323 (NG_000007.3, 54132–54133) to probe 11980-L12803 (NG_000007.3, -) was found, indicating a deletion of ∼50 kb from the β-globin cluster (Figure 8). This rare ^G^γ(^A^γδβ)^0^-thalassemia mutation was not identified in Chinese before.

### The Future Health Burden Prediction

On average, the prevalence is 4.13% (95% confidence interval (CI), 3.20% to 5.07%) for β-thalassemia, 5.22% (95% CI, 4.18% to 6.27%) for α-thalassemia caused by the Southeast Asian deletion (–SEA), and 1.89% (95% CI, 1.25% to 2.53%) for α-thalassemia caused by a single α-globin gene deletion or mutation in the Meizhou region. According to the population report of the Meizhou government in 2011, the annual birth, annual birth rate and natural growth rate were 58,000/year, 11.23 ‰ and 5.64‰, respectively. The estimated numbers of pregnancies each year in the Meizhou region in which the fetus would be at risk for β-thalassemia major or intermedia, Bart’s hydrops fetalis, and Hb H disease are 25 (95% CI, 15 to 38), 40 (95% CI, 26 to 57), and 15 (95% CI, 8 to 23), respectively.

## Discussion

This is the first molecular epidemiological survey of hemoglobinopathies in the Hakka population from eight representative geographical areas in the Meizhou region. Hakka (Hakka Han) are Han Chinese who speak the Hakka dialect and mostly inhabit in Guangdong, Jiangxi, Guangxi, Sichuan, Hunan and Fujian provinces of China. There are about 80 million Hakka in the world. The Meizhou region was the largest Hakka community. In our study, 15299 subjects attended the screening programs for hemoglobinopathies, including α/β-thalassemia, hemoglobin variants, and HPFH/δβ-thalassemia. Our results demonstrate that there is an extremely high heterozygote frequency of hemoglobinopathy in this region, especially the frequency of α/β-thalassemia. The heterozygote frequencies of α/β-thalassemia, Hb variants and HPFH/δβ-thalassemia are 11.24%, 0.477% and 0.002%, respectively.

Our study reveals a high prevalence of α/β-thalassemia in the region, which is similar to the average level of Guangdong province (11.07%, *P* = 0.842) [20] but lower than that of Guangxi Zhuang Autonomous Region (24.51%, *P* = 0.000) [6]. The heterozygote frequencies of α-thalassemia and β-thalassemia in Meizhou are 7.11% and 4.13%, respectively. The spectrum of both α and β-thalassemia mutations is similar to that previously described in southern China, such as Shenzhen [22], Guangzhou [20] and Hongkong [23]. α-thalassemia is mainly caused by the Southeast Asian deletion (–SEA), which displays normal hemoglobin or slightly microcytic hypochromic anemia, and therefore results in fetuses with hemoglobin Bart’s hydrops fetalis syndrome (absence of all four α-globin genes; commonly as –SEA/−SEA. IVSII-654 (C>T) is the most common β-thalassemia mutation, followed with CD41-42 (-TTCT), -28(A>G) and CD17 (A>T). These four mutations account for 86% (62/72) of β-thalassemia alleles in the Meizhou region, and these β-thalassemia heterozygotes shows increased counts of microcytosis.

The prevalence of hemoglobin variants in the Meizhou region reported in this study (0.477%) is lower than that reported for this area 30 years ago (0.575%) [17], but statistically insignificant between them (*P* = 0.162). Also, the prevalence of Meizhou area is similar to that of the neighboring Chaozhou area (0.358%, 41/11450), there is no significance statistically between the two areas (0.358%) (*P* = 0.133) [11]. The frequency of hemoglobin variants in the Meizhou region is higher than the average of Guangdong province (0.368%, *P*<0.05) [24], the average of 7 provinces in the southern region of the Yangtze River (0.330%, *P* = 0.002) and the average of 7 provinces in the northern region of the Yangtze River (0.171%, *P* = 0.000) [24], [25]. The frequency of hemoglobin variants in the Meizhou region is second only to Yunan province (5.94%, *P* = 0.000) in southern China [24].

The main subgroups of hemoglobin variants in the Hakka population of the Meizhou region are E (24.7%) and G/D (21.9%) group. Compared with other areas of Guangdong province, the frequency of the G/D group (0.118%, 18/15299) in the Meizhou region is higher than that of other areas (Guangzhou, Foshan, Zhanjiang) in Guangdong province (0.018%, 12/66539) (*P* = 0.000) [24], [26]. According to our knowledge, the proportion of abnormal hemoglobin of the G/D group in the northern Han population is higher than that of its southern counterpart [27]. In this study, the proportion of abnormal hemoglobin of the G/D group of the Hakka people in Meizhou is higher than that in the pearl river delta region, such as Guangzhou [26]. Therefore, we speculate that Hakka people has more genetic influence of the northern Han population than other people in Guangdong province.

Hb E is the most common hemoglobin variant in southern Asia, and is thought to spread from south to north [28], [29]. It is also one of the major hemoglobin variants in southern China, including eastern Guangdong [24]. The frequency of Hb New York in the Meizhou region is significantly higher than those of the neighboring Chaoshan and other areas of Guangdong province, consistent with previous studies [11], [17], [24]. According to previous reports [17], [24], Hb New York originated from the Hakka people, and spread to other regions in the world with the migration of Hakka people. Previous studies also suggested that Hb Q-Thailand is mainly distributed in the Dabu and Fengshun counties [17], [27]. We demonstrate that Hb Q-Thailand is evenly distributed in the Meizhou region. We hypothesize that the disappearance of uneven distribution of the characteristics among different regions resulted from population migration. Hb E heterozygotes often display microcytosis, without apparent anemia. Hb Q-Thailand is linked with the 4.2 kb deletion in our study. Therefore, these people show slight microcytosis. The other hemoglobin variants shows normal red blood cell parameters.

To date, only 4 kinds of HPFH have been found in mainland China, partly owing to the lack of diagnostic technology. A few of HPFH cases found in hemoglobinopathies screening or clinical hemoglobin electrophoresis were identified [6]. Southeast Asian (Vietnamese) deletion HPFH, which was first found in two unrelated individuals of Vietnamese background by Motum PI in 1993 [14], [30], is the commonest genotype in southern China. In China, it was first identified by Xu XM in 1998 from two cases of Cantonese. 2 cases of Southeast Asian deletion HPFH are identified by MLPA and Gap-PCR in our survey. Moreover, a case of Belgian^ G^γ(^A^γδβ)^0^ thalassemia is found in this screening, a first report of this type in Chinese. Although Southeast Asian deletion HPFH heterozygotes and Belgian^ G^γ(^A^γδβ)^0^ thalassemia are asymptomatic, when they are combined with β-thalassemia, they can lead to β-thalassemia major or intermedia symptom. Therefore, genetic counseling and prenatal diagnosis are absolutely necessary in the couple carriers of HPFH/^G^γ(^A^γδβ)^0^ thalassemia and β-thalassemia.

On the basis of our epidemiological results, thalassemia is a large public health problem in southern China. Meizhou is a poverty-stricken mountainous region in eastern Guangdong. The annual per capita disposable income of the urban residents and the annual per capita net income of rural residents are only ¥13,113/$2,059 and ¥5,390/$846, respectively (Data from the Webpage of the Meizhou government (http://www.meizhou.gov.cn). Children with β-thalassemia major need regular blood transfusions, iron-chelation therapy with deferoxamine and even hematopoietic stem cell transplantation. The annual cost of blood transfusions and deferoxamine treatments for one patient with β-thalassemia major is estimated to be about ¥100,000/$15,690 in China. The huge gap of income and medical expenses makes many families extremely poor. Therefore, it needs the local government and the health departments to develop a system of prevention and control program to decrease hemoglobinopathies in the Meizhou region. Our recommendations are as follows: (1) medical workers and health administrations should participate in the hemoglobinopathy prevention program. At the same time, new related policies and laws should be introduced and carried out to prevent hemoglobinopathy and help the families with thalassemia major or intermedia; (2) prevention network of hemoglobinopathy should be established in the region, including establishment of a hemoglobinopathy database, hematological analysis laboratories, genetic counselling clinics, prenatal diagnosis centers and neonatal screening centers; (3) relevant hereditary disease information about thalassemia should be made readily available for the medical staffs and the general population.

### Conclusions

Our results provide a detailed prevalence and molecular characterization of hemoglobinopathies in Hakka people of the Meizhou region. We demonstrate that there is an extremely high heterozygote frequency of hemoglobinopathy in this region. Therefore, genetic counseling and prenatal diagnosis are absolutely necessary in this region. There are 10 hemoglobin variants in the Hakka population. Hb Q-Thailand, linked with the 4.2 kb deletion and Hb E, show slight red cell microcytosis, the other 8 hemoglobin variants are clinically insignificant, and we firstly identify a rare case of Hb J-Broussais (Hb Tagawa-I) and a case of Belgian^ G^γ(^A^γδβ)^0^–thalassemia in Chinese.
